# Lipidomic predictors of residual insulin production in adults with newly diagnosed type 1 diabetes

**DOI:** 10.1007/s11306-026-02465-x

**Published:** 2026-06-17

**Authors:** Pernille Emilie Hostrup, Karolina Sulek, Flemming Pociot

**Affiliations:** 1 Department of Clinical Research, Copenhagen University Hospital – Steno Diabetes Center Copenhagen, Herlev, Denmark; 2 Novo Nordisk A/S, Bagsvaerd, Denmark; 3 Department of Clinical Medicine, Faculty of Health and Medical Sciences, University of Copenhagen, Copenhagen N, Denmark; 4 Copenhagen University Hospital – Steno Diabetes Center Copenhagen, Herlev, Denmark

**Keywords:** Beta cell function, Newly diagnosed type 1 diabetes, Clinical trial, Lipidomic, Remission phase, Adult-onset type 1 diabetes

## Abstract

**Introduction:**

Alterations in the lipidomic profile have been implicated in the development and disease progression in type 1 diabetes. We have previously demonstrated that the lipidomic profile at the time of diagnosis could predict residual insulin production the following 6 to 12 months, in children.

**Objectives:**

Given the strong influence of age on residual insulin production, we aimed to investigate the association between the lipidomic profile and residual insulin production in adults newly diagnosed with type 1 diabetes.

**Method:**

We performed lipidomic profiling of plasma samples from 29 adults newly diagnosed with type 1 diabetes. Linear regression and backward stepwise multiple regression analyses were used to assess the association between the lipidomic profile and C-peptide levels during a 2 h mixed-meal tolerance test. All data was derived from an original randomised clinical trial with the trial registration number: EudraCT number: 2019-004434-41.

**Results:**

The lysophosphatidylcholines (LPCs) were the strongest predictors of residual insulin production. Higher baseline LPC levels were associated with increased residual insulin production at week 52. Specifically, LPC(20:4/0:0), LPC(0:0/20:4), and LPC(0:0/16:0) were significantly associated with improved residual insulin production. Additionally, reduced levels of sphingolipids, including sulfatides, were negatively associated with residual insulin production.

**Conclusion:**

In adults, the LPC class, in particular, showed potential as a biomarker for preserved residual insulin production. This contrasts previous observations in children and highlights the importance of age-stratified research to understand the role of the lipidome in type 1 diabetes.

## Introduction

Type 1 diabetes is an autoimmune disease characterised by an immune-mediated destruction of pancreatic insulin-producing beta cells, resulting in insulin deficiency and hyperglycaemia (DiMeglio et al., 2018). Typically, 80–90% of the beta cell mass is lost at the time of diagnosis (Gepts, 1965). Nevertheless, around 40% of individuals with newly diagnosed type 1 diabetes experience a transient phase of increased endogenous insulin production shortly after initiating insulin therapy (Zhong et al., 2020). This period referred to as the remission phase, is dominated by a reduced need for exogenous insulin while maintaining glycaemic control and is often regarded as a “window of opportunity” for interventional trials aimed at preserving beta cell function. However, despite extensive efforts, only a few clinical trials have demonstrated significant improvements in beta cell function and with uncertain clinical relevance (Forlenza et al., 2023; Quattrin et al., 2020; Ramos et al., 2023) .

One challenge in achieving success in these trials resides in the inherent heterogeneity of type 1 diabetes. Some individuals experience a rapid progression with a negligible remission phase, whereas others remain in remission for months or even years (Humphreys et al., 2019). Therefore, early stratification based on the anticipated lengths of the remission phase would benefit both healthcare providers, by enabling personalised treatment, and researchers, by identifying individuals most likely to benefit from a given intervention. Factors such as age at disease onset, presence of ketoacidosis at disease debut, and sex have been proposed as predictors of the remission phase (Zhong et al., 2020). However, reliable biomarkers to predict the remission phase remain elusive.

Emerging data have revealed notable alterations in the plasma lipid profile around the time of type 1 diabetes diagnosis. But whether such alterations reflect a general metabolic shift associated with the disease or play a role in the pathogenesis of type 1 diabetes remains debatable. Longitudinal trials have identified some lipidomic biomarkers with the potential to predict the risk of type 1 diabetes in high-risk individuals (Lamichhane et al., 2018, 2019). However, most of these studies focused on the pre-diagnosis period and only a few investigated the remission phase (Overgaard et al., 2018). Moreover, most biomarker studies are conducted in paediatric cohorts despite adult-onset type 1 diabetes being the most prevalent form of the disease.

In this study, we investigated the relationship between the plasma lipidome at disease onset and residual insulin production during the first year following diagnosis in adults newly diagnosed with type 1 diabetes. We aimed to discover potential biomarkers for preserved residual insulin production and explore the impact of specific lipid species on the remission phase.

## Method

### Study participants

Data for this study were derived from our phase 2, placebo-controlled, double-blinded trial conducted at Steno Diabetes Center Copenhagen from 2020 to 2022 (Hostrup et al., 2025). The original study included 58 individuals diagnosed with type 1 diabetes no more than 6 weeks prior to randomisation. Participants were allocated in a 1:1 ratio to receive the active drug, fenofibrate, or placebo for 52 weeks, and followed with regular study visits in the period. As fenofibrate is a lipid-manipulating drug, we only included the placebo group in the present study. These individuals received standard care, making them representative of the adult-onset type 1 diabetes population in Denmark. Of the 29 individuals randomised to placebo, 27 completed the full 52-week intervention and were included in the statistical analysis (*N* = 27). We refer the readers to the original study for details regarding inclusion criteria, randomisation, and follow-up visits. The trial protocol received ethical approval from local authorities under the EudraCT no.: 2019-004434-41 and was conducted under the principles of the Declaration of Helsinki. Informed consent was obtained from all participants following the provision of comprehensive oral and written information.

### Assessment of residual insulin production

Residual insulin production was evaluated by measuring plasma C-peptide levels during a 2-hour mixed meal tolerance test (MMTT) at baseline and 12, 26, and 52 weeks after enrolment. After an overnight fast, participants consumed a standardised meal, without exogenous insulin administration. Venous blood samples were collected before ingestion and at 15, 30-, 60-, 90-, and 120 min post-ingestion. C-peptide and glucose levels were measured immediately following each time point. The full study protocol can be found in the original paper (Hostrup et al., 2025).

### Lipidomic profiling

Lipidomic profiling was conducted on venous blood samples collected at baseline, prior to the MMTT, following an 8-hour fast. As previously described, 10 µL of plasma was mixed with 10 µL of 0.9% w/v NaCl (aq) and 120 µL of chloroform/methanol (2:1) containing internal standards (Hostrup et al., 2025). Lipids were extracted into the chloroform phase and analysed using an Agilent ultra-high performance liquid chromatography system coupled with a quadrupole time-of-flight mass spectrometer (UHPLC-QTOFMS, Agilent). Samples were processed in a randomised order, with quality control pooled plasma samples inserted at regular intervals throughout the run (*n* = 10, for both positive and negative ionisation modes). Lipidomic data were pre-processed using Skyline software, with peak identification and matching conducted against an in-house library, validated across 31 European laboratories using the National Institute of Standards and Technology Standard Reference Material for human plasma metabolites. Data were normalised to internal standards and corrected for batch effects before statistical analysis. Missing values were imputed using the K-Nearest Neighbours algorithm in R.

### Sample size and statistical analysis

As the present study used data derived from the parent phase 2 randomised trial (Hostrup et al., 2025), no new a priori sample size calculation was performed for the present aim, and the study is not formally powered to detect small effects.

Prior to statistical analysis, lipidomic data were log10-transformed and normalised to the interquartile range. Data normality was assessed using Q-Q plots. Unless otherwise specified, data are presented as the predicted change in the dependent variable for each unit increase in an explanatory variable, with all covariates held constant, and are reported with corresponding 95% confidence intervals. Linear regression models were employed to investigate the association between lipid profiles and changes in C-peptide levels over time. Covariates included in the models were age at disease onset, sex, HbA1c, and stimulated C-peptide level at baseline. Pearson’s correlation coefficient was used in an exploratory analysis to assess linear relationships between individual lipid species and changes in C-peptide levels. Lipid species that significantly correlated with changes in C-peptide were further analysed using linear regression as described above. Backward stepwise multiple linear regression was used to identify the most significant predictors of changes in residual insulin production. Lipid classes and pre-specified covariates (age, sex, HbA1c, and baseline C-peptide) were included as independent variables in the model. Results from the backward multiple regression analysis are presented as adjusted R-squared values along with corresponding ANOVA results. All p values are two-sided, with a significance level set at 0.05. The Benjamini-Hochberg procedure was applied to control for type I errors. Statistical analyses were performed using IBM^®^ SPSS^®^ Statistics version 25.0.

## Results

### Study participants

**Fig. 1:**
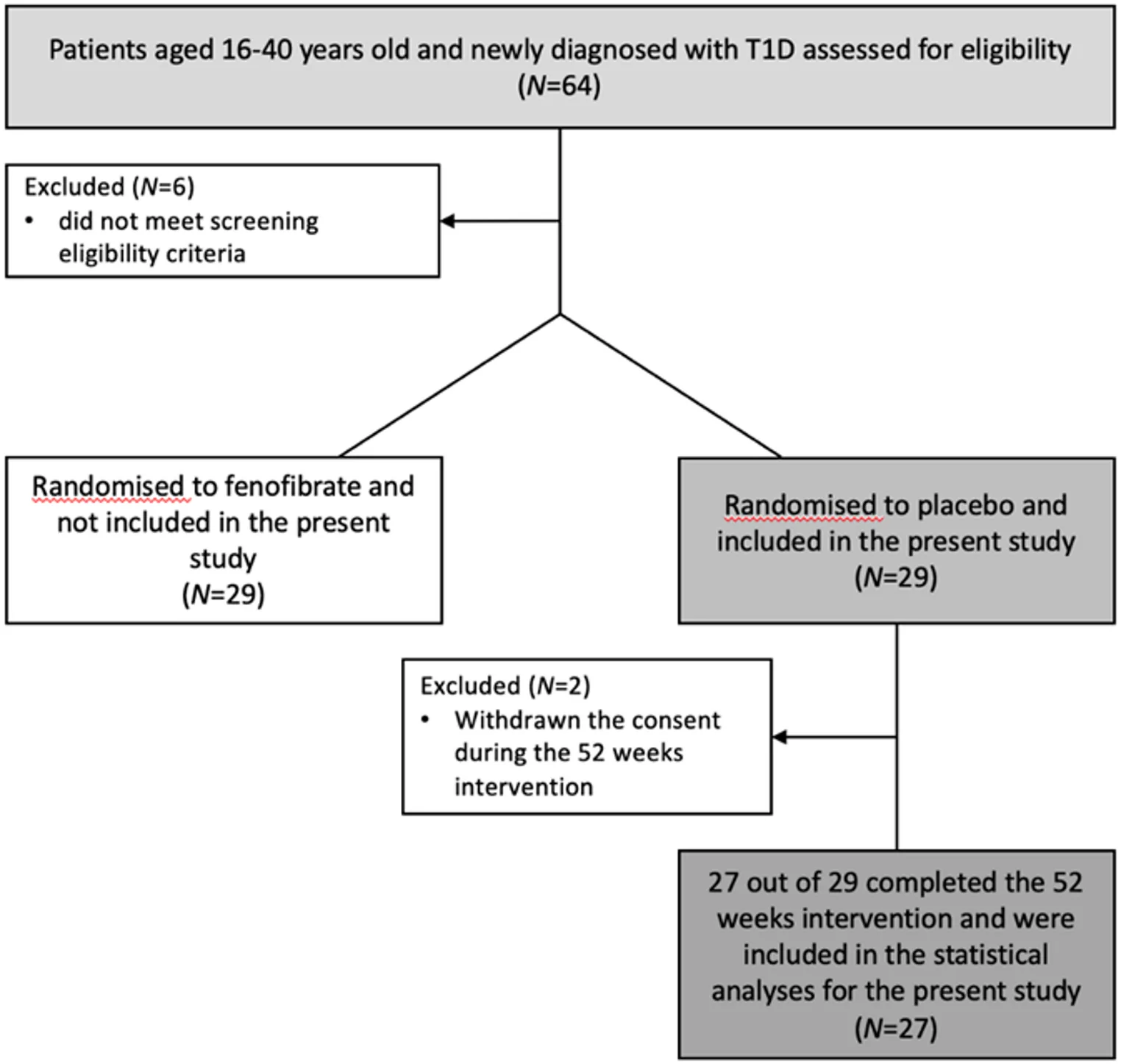
Participant flow diagram. Sixty-four patients age 16–40 years newly diagnosed with type 1 diabetes were assessed for eligibility for the original study. Fifty-eight were randomised to either the active arm receiving fenofibrate (not included in the present study) and 29 to placebo (included in the present study). During the 52-week intervention, two participants in the placebo arm withdrew consent and were excluded from the analysis. Twenty-seven of 29 placebo-randomised participants completed the 52-week intervention and were included in the statistical analyses of the present study (*N* = 27)

Twenty-seven out of 29 participants completed the full 52-week study period and were included in the statistical analysis for the present study (see Fig. 1). The clinical and anthropometric characteristics of these 27 participants are summarised in Table 1. The mean age at enrolment was 28 ± 6 years, with an average disease duration of 24 ± 11 days from diagnosis to the baseline visit. Nineteen participants (70%) experienced a period of partial remission during the 52-week follow-up, defined as an HbA1c < 53 mmol/mol (7.0%) and an insulin requirement of ≤ 0.4 U/kg body weight.

**Table 1:** Clinical characteristics during 52 weeks

Time from baseline	0 weeks (baseline)	12 weeks	26 weeks	52 weeks
Characteristics	Outcome ± SD	Outcome ± SD	Outcome ± SD	Outcome ± SD
Male sex, No. (%)	17 (63)	NA	NA	NA
Age, years	28 ± 6	NA	NA	NA
Body mass index, kg/m^2^	22.5 ± 2.4	23.0 ± 1.9	23.0 ± 2.1	23.1 ± 2.3
HbA1c, mmol/mol	91.9 ± 21.4	51 ± 12	49 ± 12	50 ± 9
Insulin use, U kg^1^ day^1^	0.28 ± 0.17	0.22 ± 0.13	0.22 ± 0.14	0.27 ± 0.16
In partial remission, No.	0 (0)	15 (56)	16 (59)	15(56)
C-peptide level^a^, pmol/L	645 ± 322	760 ± 288	709 ± 337	606 ± 299
Disease duration^b^, days	24 ± 11	NA	NA	NA
Type 1 diabetes-specific autoantibodies^c^, No.	3 ± 1	NA	NA	NA
Minimum	1			
Maximum	4			

^a^C-peptide measurements are presented as the area under the curve values after a 2-hour MMTT

^b^Number of days from diagnosis to the baseline visit

^c^Defined as anti-glutamic acid decarboxylase 65, anti-islet antigen 2, anti-insulin, and zinc transporter 8

### Lipid classes associated with residual insulin production

In plasma samples collected at baseline, we identified 511 individual lipids representing 19 distinct lipid classes (Hostrup et al., 2025). We analysed the sum values of each lipid class using linear regression, with changes in C-peptide levels at each time point as the dependent variable (see Table 2). After adjusting for age, sex, HbA1c, and stimulated C-peptide at baseline, only the lipid class of lysophosphatidylcholines (LPCs) emerged as a significant predictor of changes in C-peptide levels during the first year after diagnosis. Specifically, higher baseline LPC levels were associated with increased C-peptide levels at week 52 (predicted change in C-peptide level: 130 pmol/L [95% CI 10 to 243], *p* < 0.05). However, after controlling the false discovery rate (FDR) at 5%, this association did not retain statistical significance.

**Table 2:** Association between lipid classes at baseline and change in C-peptide level during 52 weeks

Time from baseline	12 weeks			26 weeks			52 weeks		
Lipid classes	Change in C-peptide pmol/L^a^ (95% CI)		*p* value^b^ (adjusted)	Change in C-peptide pmol/L^a^ (95% CI)		*p* value^b^ (adjusted)	Change in C-peptide pmol/L^a^ (95% CI)		*p* value^b^ (adjusted)
Ceramide	16	(− 92, 124)	0.92 (0.77)	− 49	(− 153, 55)	0.96 (0.34)	57	(− 38, 152)	0.42 (0.13)
Cholesterol Ester	5	(− 95, 105)	0.92 (0.92)	− 22	(− 120, 76)	0.96 (0.64)	65	(− 22, 151)	0.42 (0.22)
Diglyceride	71	(− 105, 247)	0.92 (0.41)	− 20	(− 196, 155)	0.96 (0.81)	49	(− 113, 210)	0.64 (0.54)
Dimethyl-phosphatidyl-ethanolamine	− 7	(− 139, 126)	0.92 (0.92)	3	(− 133, 127)	0.96 (0.96)	87	(− 27, 201)	0.42 (0.13)
Fatty acid	40	(− 77, 157)	0.92 (0.49)	61	(− 52, 174)	0.96 (0.27)	23	(− 84, 130)	0.74 (0.66)
Hexosylceramide	35	(− 75, 145)	0.92 (0.52)	− 6	(− 115, 104)	0.96 (0.92)	40	(− 60, 139)	0.61 (0.42)
Lactosylceramide	85	(− 22, 191)	0.92 (0.11)	17	(− 94, 127)	0.96 (0.76)	15	(− 88, 117)	0.78 (0.77)
Lysodimethyl-phosphatidyl-ethanolamine	90	(− 25, 205)	0.92 (0.12)	62	(− 55, 179)	0.96 (0.28)	82	(− 23, 186)	0.42 (0.12)
**Lysophosphatidyl-choline**	79	(− 57, 215)	0.92 (0.24)	70	(− 64, 204)	0.96 (0.29)	**130**	**(16**,** 243)**	0.42 (0.03*)
Lysophosphatidyl-ethanolamine	35	(− 87, 156)	0.92 (0.56)	47	(− 71, 166)	0.96 (0.41)	87	(− 17, 191)	0.42 (0.10)
Phosphatidic Acid	85	(− 58, 228)	0.92 (0.23)	− 6	(− 151, 140)	0.96 (0.94)	69	(− 62, 199)	0.45 (0.29)
Phosphatidyl-choline	13	(− 102, 129)	0.92 (0.81)	− 24	(− 137, 89)	0.96 (0.67)	68	(− 33, 168)	0.42 (0.18)
Phosphatidyl-ethanolamine	17	(− 122, 155)	0.92 (0.81)	19	(− 117, 154)	0.96 (0.78)	74	(− 48, 195)	0.42 (0.22)
Phosphatidyl-glycerol	9	(− 121, 140)	0.92 (0.88)	− 15	(− 143, 114)	0.96 (0.82)	67	(− 48, 181)	0.42 (0.21)
Phosphatidyl-inositol	− 9	(− 142, 125)	0.92 (0.89)	− 55	(− 184, 74)	0.96 (0.39)	17	(− 105, 138)	0.78 (0.78)
Phosphatidyl-serine	− 9	(− 145, 127)	0.92 (0.89)	− 3	(− 130, 137)	0.96 (0.96)	44	(− 75, 163)	0.42 (0.09)
Sphingomyelin	13	(− 114, 141)	0.92 (0.83)	45	(− 168, 79)	0.96 (0.46)	60	(− 53, 173)	0.45 (0.28)
Sulfoglycoshingo-lipid	− 62	(− 191, 68)	0.92 (0.33)	− 15	(− 145, 115)	0.96 (0.82)	17	(− 105, 138)	0.61 (0.45)
Triglyceride	54	(− 69, 178)	1.00 (0.37)	9	(− 115, 132)	0.96 (0.88)	38	(− 75, 151)	0.62 (0.49)

Values shown in bold are of particular interest and are addressed further in the text

^a^Predicted change in C-peptide levels associated with lipid levels at baseline. C-peptide measurements are presented as the area under the curve values after a 2 h MMTT

^b^Raw and Benjamini-Hochberg adjustment *p* values (x) with a false discovery rate at 5%

*p ≤ 0.05

To assess the combined effect of different lipid classes, we conducted a backward multiple regression analysis with stepwise elimination of lipid classes (see Table 3). This approach identified nine models that significantly predicted changes in C-peptide levels at week 52. The best-fitting model explained 77% of the variance in C-peptide levels (adjusted R²: 0.77, F: 8.4, *p* < 0.001) and included cholesterol esters (CEs), diacylglycerols (DGs), LPCs, lysophosphatidylethanolamines (LPEs), phosphatidylcholines (PCs), phosphatidylglycerols (PGs), sphingomyelins (SMs), dimethylphosphatidylethanolamines (dMePes), and phosphatidylinositols (PIs). Notably, LPCs consistently emerged as the most significant predictor across all nine models.

**Table 3:** Best-fitted models to predict change in C-peptide level at week 52

Models^a^	1	2	3	4*	5	6	7	8	9
R^2, b^ (*p* values)	0.77 (2.2E-02)	0.80 (8.6E-03)	0.82 (3.4E-03)	0.83 (1.5E-03)	0.82 (7.4E-04)	0.80 (7.3E-04)	0.78 (5.4E-04)	0.77 (3.7E-04)	0.77 (1.8E-04)

Lipid classes	The significant level of the independent lipid class (*p* values of the coefficients) in each model								
Ceramide	Exc.	Exc.	Exc.	Exc.	Exc.	Exc.	Exc.	Exc.	Exc.
Cholesterol Ester	8.4E-03	2.2E-03	1.1E-3	6.5E-04	4.6E-04	8.6E-04	5.8E-04	8.2E-04	6.2E-04
Diglyceride	1.6E-01	6.0E-2	4.0E-02	3.6E-02	4.7E-02	6.6E-02	5.2E-02	1.0E-02	1.1E-02
Dimethyl-phosphatidyl-ethanolamine	5.3E-3	5.5E-04	1.9E-04	9.3E-05	5.5E-05	8.0E-05	9.6E-05	2.2E-05	1.6E-05
Fatty Acid	7.5E-01	Exc.	Exc.	Exc.	Exc.	Exc.	Exc.	Exc.	Exc.
Hexosylceramide	4.7E-01	4.2E-01	3.4E-01	3.4E-01	Exc.	Exc.	Exc.	Exc.	Exc.
Lactosylceramide	1.1E-01	4.2E-02	3.4E-02	3.1E-02	3.5E-02	1.2E-01	2.2E-01	Exc.	Exc.
Lysodimethyl-phosphatidyl-ethanolamine	5.6E-1	5.9E-01	Exc.	Exc.	Exc.	Exc.	Exc.	Exc.	Exc.
**Lysophosphatidyl-choline**	**7.0E-04**	**2.4E-04**	**9.6E-05**	**3.1E-05**	**1.5E-05**	**2.0E-05**	**9.5E-06**	**9.4E-06**	**6.4E-06**
Lysophosphatidyl-ethanolamine	1.8E-03	6.8E-04	2.1E-04	6.5E-05	3.8E-05	5.4E-05	2.2E-05	2.3E-05	1.6E-05
Phosphatidic Acid	5.2E-01	5.5E-01	4.5E-01	Exc.	Exc.	Exc.	Exc.	Exc.	Exc.
Phosphatidylcholine	1.4E-02	2.5E-03	1.3E-03	9.1E-04	7.8E-04	1.0E-03	1.3E-03	1.3E-03	1.1E-04
Phosphatidyl-ethanolamine	8.2E-02	6.1E-02	5.1E-02	4.4E-02	6.0E-02	1.3E-01	1.8E-01	3.6E-01	1.3E-04
Phosphatidylglycerol	4.1E-03	7.1E-04	3.4E-04	1.9E-04	1.0E-04	1.5E-04	1.9E-04	1.7E-04	Exc.
Phosphatidyl-inositol	1.62E-01	1.17E-01	8.7E-02	8.5E-02	1.3E-02	1.9E-02	7.6E-03	2.6E-03	2.1E-04
Phosphatidylserine	1.89E-01	1.28E-01	1.2E-01	1.2E-01	1.3E-01	Exc.	Exc.	Exc.	Exc.
Sphingomyelin	5.27E-03	2.15E-03	1.1E-03	6.7E-04	3.7E-04	5.4E-04	6.9E-04	9.7E-04	3.2E-04
Sulfoglyco-shingolipid	1.72E-01	1.18E-01	9.2E-02	9.9E-02	1.2E-01	2.0E-01	Exc.	Exc.	Exc.
Triglyceride	Exc.	Exc.	Exc.	Exc.	Exc.	Exc.	Exc.	Exc.	Exc.

Values shown in bold are of particular interest and are addressed further in the text

^a^By multiple backward regression with a probability of F at 0.05 for entry in the model and 0.10 for removal, nine different models could significantly explain the change in C-peptide at week 52. Variables contributing the least to the explanatory power were stepwise removed from the model (Exc.)

*Model 4 represent the best-performing model with R^2^ 0.83

### LPC species associated with residual insulin production

Given that the LPC lipid class was the strongest predictor of changes in C-peptide levels at week 52, we further investigated the relationship between individual LPC species at baseline and changes in C-peptide levels at week 52 (see Table 4). Ten out of 45 individual LPC species were significantly correlated with increased C-peptide levels at week 52, using Pearson’s correlation coefficient, and were further analysed by linear regression, adjusted for age at disease onset, sex, HbA1c, and stimulated C-peptide level at baseline. With an FDR of 5%, LPC(20:4/0:0), LPC(0:0/20:4), and LPC(0:0/16:0) remained significant predictors of changes in C-peptide levels, with higher baseline levels of lipids associated with increased C-peptide levels at week 52.

**Table 4:** Single LPC lipids at baseline associated with change in C-peptide level at week 52

Analysis	Person’s correlation analysis			Linear regression		
Lipid species	R square^a^ (95% CI)		*p* value^b^ (adjusted)	Change in C-peptide pmol/L^c^ (95% CI)		*p* value^b^ (adjusted)
**LPC(20:4/0:0)**	0.33	(0.06, 0.61)	0.03(0.002)	**173**	**(81**,** 265)**	0.01** (0.001)
LPC(0:0/18:2)	0.31	(0.05, 0.60)	0.03 (0.002)	129	(− 2, 259)	0.09 (0.05)
LPC(18:2/0:0)	0.31	(0.05, 0.60)	0.03 (0.003)	127	(− 4, 258)	0.09 (0.06)
LPC(18:2)	0.31	(0.05, 0.60)	0.03 (0.003)	120	(− 3, 243)	0.09 (0.06)
**LPC(0:0/20:4)**	0.29	(0.04, 0.58)	0.03 (0.004)	**162**	**(42**,** 282)**	0.05* (0.01)
LPC(0:0/18:1)	0.26	(0.03, 0.55)	0.04(0.007)	75	(− 12, 163)	0.11 (0.09)
LPC(18:1/0:0)	0.26	(0.03, 0.55)	0.04 (0.007)	75	(− 12, 163)	0.11 (0.09)
LPC(18:1)	0.25	(0.02, 0.55)	0.04 (0.007)	74	(− 15, 162)	0.11 (0.10)
LPC(0:0/20:5)	0.24	(0.02, 0.54)	0.05 (0.010)	84	(− 35, 202)	0.16 (0.16)
**LPC(0:0/16:0)**	0.24	(0.02, 0.53)	0.05 (0.010)	**137**	**(31**,** 243)**	0.05* (0.01)

Values shown in bold are of particular interest and are addressed further in the text

^a^R square values calculated from Pearson’s correlation coefficient (r) between single LPCs and change in C-peptide at week 52

^b^Raw and Benjamini-Hochberg adjustment *p* values (x) with a False Discovery Rate at 5%

^c^Predicted change in C-peptide levels associated with lipid levels at baseline. C-peptide measurements are presented as the area under the curve values after a 2-hour MMTT

LPC species are denoted by fatty acyl composition (e.g., LPC 24:0/0:0 indicated a 24-carbon saturated fatty acid with no second acyl chain)

*p ≤ 0.05, **p ≤ 0.01

### Sphingolipids associated with residual insulin production

Previous studies have implicated low sphingolipid expression in the pathogenesis of type 1 diabetes (Gurgul-Convey, 2020). For this reason, manipulation of specific sphingolipids has been proposed as a potential therapeutic target in newly onset type 1 diabetes(Holm et al., 2018, 2019). To further explore this, we examined the relationship between changes in plasma sphingolipid levels and changes in C-peptide levels over 52 weeks (see Table 5). We detected five distinct sphingolipid classes, for which we observed a negative correlation between changes in sphingolipid levels and changes in C-peptide levels. However, the association was only statistically significant for hexosylceramides (HexCers) and sulfatides (SHexCers). Specifically, a one-unit increase in HexCer or SHexCer was associated with a reduction in C-peptide levels of 19 pmol/L (95% CI 36 to 3) and 51 pmol/L (95% CI 84 to 18), respectively (*p* < 0.05).

**Table 5:** Association between change in sphingolipids and change in C-peptide level at week 52

Lipid classes	Change in C-peptide pmol/L^a^ (95% CI)		*p* value^b^ (adjusted)
Ceramides	− 3	(− 8, 1)	0.19 (0.15)
**Hexosylceramides**	**− 19**	**(− 36**,** − 3)**	0.05* (0.02)
Lactosylceramides	− 20	(− 59, 18)	0.36 (0.30)
Sphingomyelin	− 1	(− 4, 1)	0.36 (0.36)
**Sulfoglycoshingolipids**	**− 51**	**(− 84**,** − 18)**	0.02* (0.003)

Values shown in bold are of particular interest and are addressed further in the text

^a^Predicted change in C-peptide levels associated with changes in lipid levels over 52 weeks. C-peptide measurements are presented as the area under the curve values after a 2-hour MMTT

^b^Raw and Benjamini-Hochberg adjustment *p* values (x) with a false discovery rate at 5%

*p ≤ 0.05, **p ≤ 0.01

## Discussion

We have previously demonstrated that the lipidome signature can predict residual beta cell function the first year after diagnosis in children with newly onset type 1 diabetes (Overgaard et al., 2018). The present study extends this work by exploring potential lipidomic markers in relation to residual insulin production within the first year after diagnosis in adults. Across 511 individual lipid species, representing 19 different lipid classes, we find that the LPC class emerged as the strongest predictor of residual insulin production 52 weeks after diagnosis in the statistical models applied. When examining individual LPS species, 3 of 43 unique lipids remained significant associated with increased residual insulin production at week 52, after adjusting for multiple comparisons.

We identified a significant association between plasma LPC levels at the time of diagnosis and residual insulin production 52 weeks later. Specifically, a one-unit increase in baseline LPC was associated with a 130 pmol/L higher C-peptide level at week 52. Although statistically significant, an effect size of 130 pmol/L is modest. This may partly reflect the relatively small change in mean C-peptide observed in the cohort over 52 weeks (645pmol/L at baseline vs. 606 pmol/L at week 52). With limited overall change in C-peptide, the ability to detect robust biomarkers is generally reduced. However, despite this modest group-level decline in C-peptide over 52 weeks there was substantial inter-individual variability in C-peptide trajectories. This variability in the cohort allowing LPC levels at baseline to capture differences in the rate of C-peptide decline at the individual level. These findings support the value of exploratory studies, such as the present, to identify biomarker that may predict C-peptide trajectories in a heterogeneous population characteristic of type 1 diabetes.

Given that the present study was conducted in adults, the limited decline in C-peptide over 52 weeks is not unexpected, as adult-onset type 1 diabetes is typically characterized by a longer remission phase. Unfortunately, we were unable to include longer-term follow-up data, as there was substantial loss to follow-up in the original trial – particularly in the placebo group from which data for the present study is derived. It could by speculated, that a longer follow-up period could enhance the predictive values of LPC, hence a greater decline in C-peptide. However, this remains hypothetical.

The observation that higher LPS values at baseline was significantly associated with higher C-peptide levels 52 weeks following diagnosis, contrasts our earlier study in a paediatric cohort, where 24 lipid species demonstrated a negative correlation with stimulated C-peptide levels 6 months after diagnosis, primarily involving the lipid classes triacylglycerols and diacylglycerols. Regarding the LPC class, no association with residual insulin production over time was observed in the paediatric cohort. However, the single LPC species, LPC(20:2) and LPC(22:5) showed a negative correlation with baseline C-peptide levels in the children – an association that was not observed in the present study conducted in adults. (Overgaard et al., 2018). Differences between adult and childhood-onset type 1 diabetes may partly explain these discrepancies. It is well-documented that adult-onset and childhood-onset type 1 diabetes exhibit distinct phenotypic differences. Younger age at diagnosis is associated with a more rapid disease progression, a shorter remission phase, and a lower likelihood of persisting residual beta-cell function in long-standing type 1 diabetes (Davis et al., 2015; Humphreys et al., 2019). This interpretation aligns with findings from an observational study on the lipidome in children at high risk for type 1 diabetes, where age was a stronger determinant of lipidomic profiling than both sex and disease status (e.g., non-progressors, progression to autoantibody-positive status without type 1 diabetes, and progression to type 1 diabetes) (Lamichhane et al., 2018). Our findings underline the importance of interpreting biomarker discovery studies within the context of the age group representing the cohort. Lastly, the existing literature provides no consistent pattern of lipid upregulation vs. downregulation concerning diabetes outcomes. For instance, in the Type 1 Diabetes Prediction and Prevention (DIPP) study progression to type 1 diabetes was associated with upregulation of some LPC lipids, whereas others were downregulated (Lamichhane et al., 2018).

Among the 19 different lipid classes identified in the present study, the LPC class exhibited the strongest association with residual insulin production, with a generally positive correlation. This observation raises the question of whether LPCs exert a protective effect on beta cell function in adults with newly diagnosed type 1 diabetes. LPCs are derived from PCs mediated by phospholipase A2 and are generally considered pro-inflammatory lipids. However, recent clinical lipidomic studies have been conflicting, with several studies showing low LPC levels in various diseases (e.g., cardiovascular diseases and Alzheimer’s disease) (Law et al., 2019). Low levels of specific LPC species have also been implicated with disease activity in type 1 diabetes. For example, LPC(20:4) and LPC(22:6) levels were significantly lower in cord-blood from individuals who later developed type 1 diabetes compared to those who became positive for diabetes-specific autoantibodies but did not progress to disease. LPC species were also generally lower in those who progressed to disease compared to healthy control, although the association did not reach significance. Interestingly, LPC(22:6) levels were higher in the autoantibody-positive group compared to healthy controls, underlining the complexity of lipid species involvement in disease progression (Lamichhane et al., 2019). While the role of LPCs in type 1 diabetes is not fully understood, our findings suggest a potential relationship between higher LPC levels and preserved beta cell function in adults; however, given the exploratory design and no validation, no causal or protective role can be inferred.

Multiple studies have demonstrated the regulatory role of sphingolipids on insulin homeostasis (Gurgul-Convey, 2020). More specifically, the glycosphingolipid sulfatide plays an important role in beta cell function. Sulfatide supports insulin folding and processing and inhibits insulin secretion by modulating the ATP-sensitive potassium channels in the beta cell plasma membrane to a more open stage (Buschard et al., 2006)—with the latter being a mechanism thought to protect against beta cell stress at rising blood glucose levels. Alterations in sulfatide expression have also been implicated in the pathogenesis of type 1 diabetes (Holm et al., 2018). While we did not observe significant association between baseline sphingolipid levels and residual insulin production over time, we identified a negative association between changes in sulfatide levels and changes in C-peptide levels the first year following diagnosis. This finding should be interpreted cautiously, but it indicates that a smaller decrease in sulfatide levels is associated with lower residual insulin production. This contract other observational studies where lower sulfatide expression has been observed in pancreatic biopsies from adult donors with newly diagnosed type 1 diabetes compared to non-diabetic controls (Holm et al., 2018). Out data, derived from plasma samples, may not fully reflect the lipidomic profile within the beta cells, which could explain the discrepant in sulfatides levels in relation to disease activity in the two studies. However, we have recently investigated the use of fenofibrate – a drug believed to upregulate sulfatide expression—in adults with newly diagnosed type 1 diabetes (Hostrup et al., 2025). Here we observed no improvement in residual insulin production in the intervention group compared to placebo, which could suggest that the relationship between sulfatide levels and beta cell function might not be straightforward.

### Strengths and limitations

Although the incidence of type 1 diabetes peaks during childhood and early adulthood, adult-onset type 1 diabetes represents the most prevalent form of the disease (Gregory et al., 2022). However, the literature is heavily skewed toward childhood-onset type 1 diabetes, with observational and interventional studies primarily focusing on younger populations. This study exclusively examined adult-onset type 1 diabetes and demonstrated that the lipidome differs from what observed in children. Our findings confirm the heterogeneity within type 1 diabetes, and results from paediatric cohorts may not directly translate to adult populations.

Nevertheless, several limitations of the present study must be emphasised. This study was conducted as a sub-study of a randomised clinical trial, originally powered to detect differences in stimulated C-peptide between an intervention group and a placebo group. Since the intervention involved a lipid-regulatory drug, only participants from the placebo group were included in the present study. Consequently, the sample size was reduced to 27 individuals, which is considerably smaller than in comparable studies; for example, our previous study in a paediatric cohort included 123 individuals (Overgaard et al., 2018). In addition, the absence of validation in the present study limits the confidence in the predictive performance of the models. The observed positive association between LPC levels and residual insulin production, should therefore be interpreted as exploratory and require replication in larger, well-powered studies.

## Conclusion

In this exploratory study, we demonstrated that lipidomic profiling at diagnosis could serve as a potential predictor of residual beta cell function in the first year after type 1 diabetes onset. The discrepancies between our findings and those reported in paediatric cohorts emphasise the complexity of lipid dynamics in type 1 diabetes. These results highlight the need for age-stratified research to further elucidate the role of the lipidome in type 1 diabetes.

## Data Availability

No datasets were generated or analysed during the current study.

## Abbreviations

CE: Cholesterol ester
DG: Diacylglycerol
dMEPe: Dimethylphosphatidylethanolamine
HexCer: Hexosylceramide
LPC: Lysophosphatidylcholine
LPE: Lysophosphatidylethanolamine
MMTT: Mixed meal tolerance test
PC: Phosphatidylcholine
PI: Phosphatidylinositol
PG: Phosphatidylglycerol
SHexCer: Sulfatide
SM: Sphingomyelin
